# Altered expression of CD226 and CD96 on natural killer cells in patients with pancreatic cancer

**DOI:** 10.18632/oncotarget.11953

**Published:** 2016-09-10

**Authors:** Yun-Peng Peng, Chun-Hua Xi, Yi Zhu, Ling-Di Yin, Ji-Shu Wei, Jing-Jing Zhang, Xin-Chun Liu, Song Guo, Yue Fu, Yi Miao

**Affiliations:** ^1^ Department of General Surgery, Pancreas Centre, First Affiliated Hospital, Nanjing Medical University, Nanjing, P.R.China; ^2^ Institute of Tumor Biology, Jiangsu Province Academy of Clinical Medicine, Nanjing, P.R.China

**Keywords:** CD226, CD96, TIGIT, NK cells, pancreatic cancer

## Abstract

The progression of pancreatic cancer (PC) is significantly associated with tumor immune escape, which may be associated with nature killer (NK) cell dysfunction. CD226, CD96, and TIGIT, which share the ligand CD155, play important roles in the regulation of NK cell function. The present study was conducted to investigate the roles of these molecules in NK cells from PC patients. Expression of these molecules on NK cells was detected from samples of 92 pancreatic cancer patients and 40 healthy controls. The expression of CD155 was also evaluated by immunohistochemistry in 88 pancreatic cancer tissues. The percentage of CD226^+^ and CD96^+^ NK cells was significantly lower in PC patients than in the healthy controls; however, the mean fluorescence intensity of CD226 and CD96 was not significantly different between the two groups. TIGIT expression on NK cells from PC patients was similar to that in the healthy controls. Additionally, the expression of CD226 was positively correlated with CD96. Further analysis demonstrated that the decrease in the percentage of CD226^+^ and CD96^+^ NK cells was associated with tumor histological grade and lymph node metastasis. Moreover, the CD155 levels in PC tissues were significantly higher than those in adjacent tissues. Our results suggest that a lower percentage of CD226^+^ and CD96^+^ NK cells may contribute to tumor immune escape in PC patients; moreover, the use of NK cells with high CD226 and CD96 expression to treat PC cells with high CD155 expression may have potential and should be explored in the future.

## INTRODUCTION

Pancreatic cancer (PC) is an extremely aggressive cancer of the digestive system, and is associated with a high mortality rate (fifth leading cause of cancer-related death) [1], low resection rate (less than 20%) [2], poor five-year survival (less than 6%) [1], and low chemotherapy sensitivity [3]. The progression of PC is significantly promoted by tumor immune escape, which results from the dysfunction of multiple immune cells, including natural killer (NK) cells and natural killer T (NKT) cells [4, 5].

NK cells are lymphocytes that serve as the first line of defense against virus-infected cells and cancer cells in the human body [6]. The function of NK cells is generally influenced by the balance of activating and inhibitory receptors on the surface of these cells, as these receptors combine with their specific ligands expressed on target cells [7]. A decrease or increase in the expression of activating or inhibitory receptors usually indicates NK cell dysfunction. NK cell dysfunction has been widely observed in different cancers, such as pancreatic cancer and gastric cancer, but the receptors involved need to be further studied [8, 9].

The DNAX accessory molecule (CD226, also known as DNAM-1) is a well investigated activating receptor on NK cells, platelets, and some subsets of T cells [10, 11]. CD226 binds with CD155 or CD112 on target cells to further enhance NK cell cytotoxicity and improve cytokine secretion [12]. CD96 (also known as TACTILE) also belongs to the activating receptor family, and it is capable of binding with CD155 to enhance adhesion between the NK cells and target cells [13]. According to previous studies, CD96 plays an important role in the cytotoxicity of NK cells; this was also found in the case of CD226 [13, 14]. TIGIT is characterized as an inhibitory receptor on NK cells and T cells. It was observed that TIGIT could suppress the function of NK cells by binding with CD155 or CD112 on target cells [15].

NK cell dysfunction caused by a decrease in CD226 expression has been reported in many malignancies, including PC, in our previous study [21–23]. However, the sample size was small; this study examines CD226 expression on NK cells in a larger population of PC patients. We have also investigated the role of two other receptors (CD96 and TIGIT) in mediating the dysfunction of NK cells in PC patients for the first time by evaluating their expression in these patients.

## RESULTS

### Expression of CD226, CD96, and TIGIT on circulating NK cells

**Figure 1:**
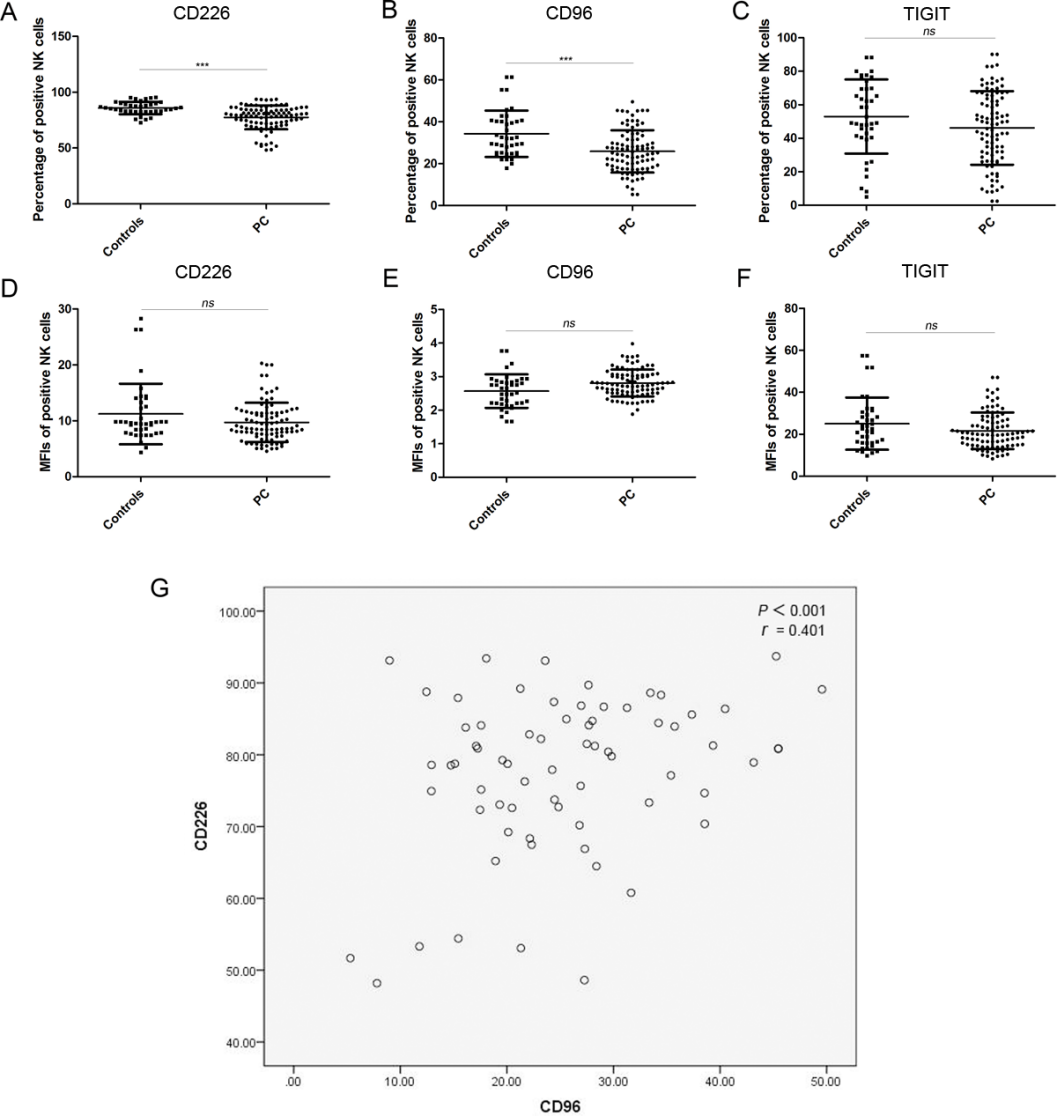
xpression of CD226, CD96, and TIGIT on NK cells. **A.**–**C.** The percentage of CD226^+^, CD96^+^, and TIGIT^+^ NK cells in healthy controls and PC patients. **D.**–**F.** The MFIs of CD226^+^, CD96^+^, and TIGIT^+^ NK cells from healthy controls and PC patients. **G.** Correlation between the percentage of CD226^+^ and the percentage of CD96^+^ NK cells in PC patients.

We detected the percentage of receptor-positive NK cells in the PC patients and healthy controls by FCM, as well as the MFIs of these receptors. As shown in Table 2 and Figure 1A-1C, the percentage of CD226^+^ and CD96^+^ NK cells (expressing the two activating receptors) was significantly lower in patients with PC than in the healthy controls; however, the percentage of TIGIT^+^ NK cells (expressing the inhibitory receptor) was not significantly different between the two groups. The MFI of CD226, CD96, and TIGIT on NK cells was similar in both groups (Figure 1D-1F).

**Table 1:** Clinicopathological characteristics of the study population (*N* = 132)

Clinicopathological characteristics			
Groups		Healthy controls	Pancreatic cancer
		*n* = 40	*n* = 92
Gender	Male	24 (60.0%)	52 (56.5%)
	Female	16 (40.0%)	40 (43.5%)
Age	Median age	56	64
	Range	26-78	40-84
AJCC Stage*	0		0(0.0%)
	I		1(1.1%)
	II		68(73.9%)
	III		0(0.0%)
	IV		23(25%)

* 2010 American Joint Committee on Cancer (AJCC).

**Table 2:** Percentage and mean fluorescence intensity of CD226+/CD96+/TIGIT+ NK cells in the study population (N = 132)

		Healthy controls	Pancreatic cancer	
		Mean±SD	Mean±SD	*P*
CD226	Percentage(%)	85.84±5.58	77.41±10.62	<0.001
	MFIs	11.22±5.41	9.72±3.51	0.195
CD96	Percentage(%)	34.31±11.06	25.86±10.12	<0.001
	MFIs	2.67±0.52	2.81±0.40	0.065
TIGIT	Percentage(%)	52.98±22.14	46.17±21.93	0.114
	MFIs	25.06±12.43	21.63±8.73	0.222

Data were expressed as means ± standard deviations (Mean ± SD).

The percentage of CD226^+^ NK cells was positively correlated with the percentage of CD96^+^ NK cells (Figure 1G). These results indicate that NK cell function in PC was decreased because of the decrease in the percentage of CD226^+^ and CD96^+^ NK cells.

### Percentage of CD96^+^ and CD226^+^ NK cells according to tumor progression indicators

The tumor sample was divided into groups according to tumor characteristics known to be indicators of cancer progression, and the percentage of CD226^+^ and CD96^+^ NK cells was compared between these groups (Table 3). First, the tumors were stratified according to the presence/absence of distant metastasis; no significant differences were observed with regard to the percentage of CD226^+^ and CD96^+^ NK cells between tumors with distant metastasis and those without distant metastasis. Then, the tumors without distant metastasis were divided according to location (head/neck and body/tail); size (≤2.5 cm and >2.5 cm); histological grade (well/moderate and poor); and the absence/presence of nerve invasion, lymph node metastasis, and blood vessel invasion. Tumors with a well/moderate histological differentiation grade had a significantly higher percentage of CD226^+^ NK cells than tumors with a poor differentiation grade. Further, tumors with lymph node metastasis had a significantly higher percentage of CD96^+^ NK cells than tumors without lymph node metastasis. No association was observed between CD96 and CD226 expression and any of the other indicators of tumor progression. These findings indicate that the expression of the activating receptors CD226 and CD96 may be associated with tumor progression.

**Table 3:** Percentage of CD226^+^ and CD96^+^ NK cells according to tumor progression indicators

Pancreatic cancer						
	NO. of patients	CD226			CD96	
		**%**	*****P*****	**%**		*****P*****
**Distant metastasis**						
Absent	69	77.57±10.97	0.549	25.76±9.62		0.649
Present	23	76.93±9.69		27.08±11.27		
**Non-metastatic Pancreatic cancer**						
	**NO. of patients**	**CD226**		**CD96**		
		**%**	*****P*****	**%**		*****P*****
**Location**						
Head/Neck	49	78.14±10.45	0.682	25.83±10.51		0.916
Body/Tail	20	76.17±12.32		25.59±7.22		
**Tumor Size**						
≤2.5cm	16	79.04±10.18	0.584	23.67±9.00		0.334
>2.5cm	53	77.12±11.25		26.39±9.79		
**Histological grade**						
Well/Moderate	31	80.75±9.69	0.017	24.10±9.24		0.232
Poor	38	74.96±11.38		26.93±10.06		
**Nerve invasion**						
Absent	12	77.78±11.08	0.728	24.95±7.17		0.918
Present	57	76.52±10.90		25.93±10.10		
**Lymph node metastasis**						
Absent	29	77.45±11.08	0.851	27.64±8.40		0.047
Present	40	77.65±11.08		23.40±9.93		
**Blood vessel invasion**						
Absent	43	78.77±10.91	0.136	25.31±9.84		0.634
Present	26	75.58±10.99		26.52±9.38		

Data were expressed as means ± standard deviations (Mean ± SD).

### Expression of CD155 in PC tissues

As described in previous articles, CD226 and/or CD96 can stimulate NK cell killing of CD155-positive target cells [7, 15]. In other words, adequate CD155 expression on PC cells is essential for CD226- and/or CD96-mediated NK cell immunity. Therefore, we used IHC to assess the expression of CD155 in 88 pancreatic cancer tissues and 33 adjacent tissues.

**Figure 2:**
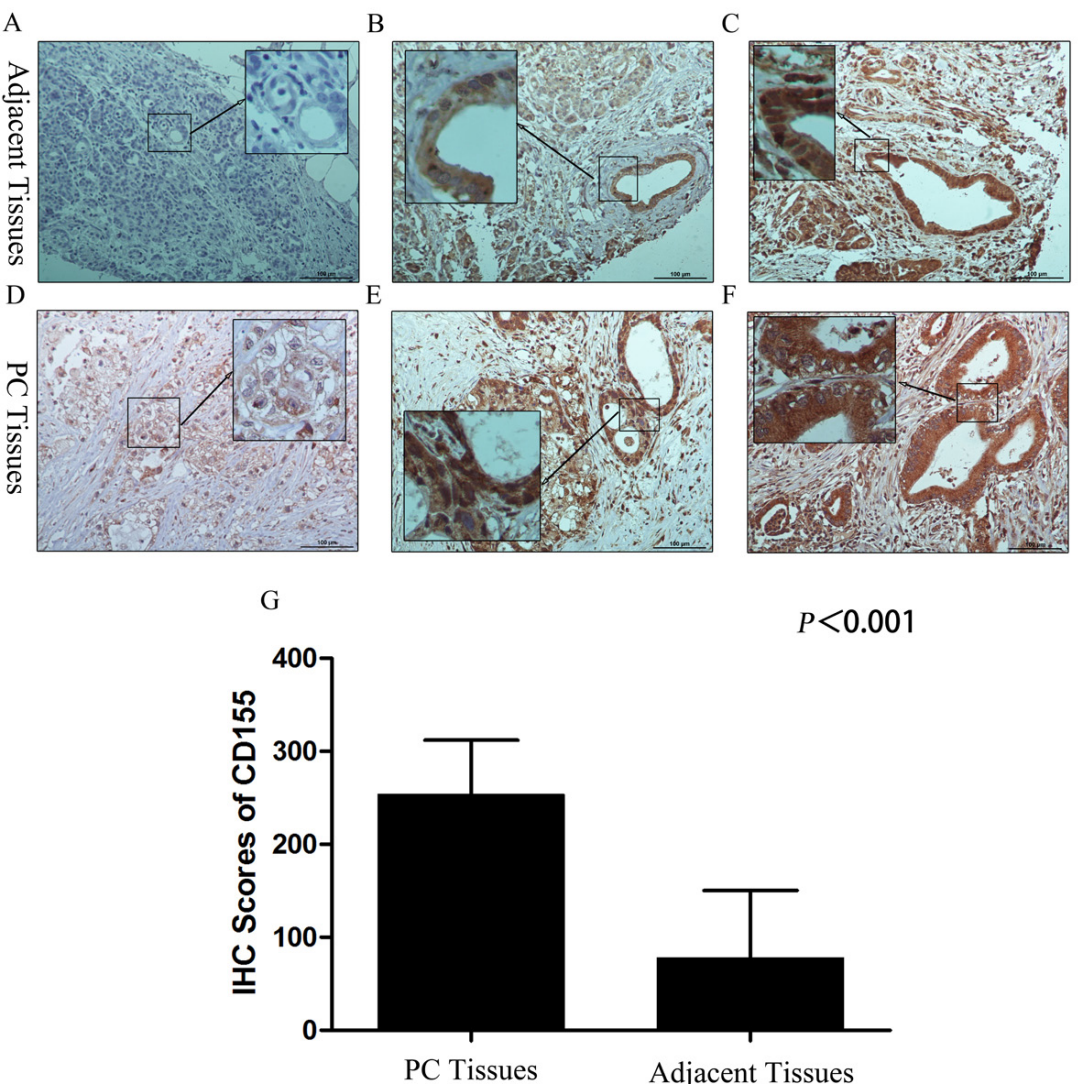
CD155 expression in pancreatic cancer and adjacent tissues **A.** Localization of CD155 was less in the cytoplasm of normal acinar cells and normal ducts. **B.** In para-cancerous tissues with a chronic pancreatitis (CP) microenvironment, CD155 was localized in the cytoplasm and membrane of the tubular cells of PanIN1-2 lesions. **C.** In para-cancerous tissues with a chronic pancreatitis (CP) microenvironment, CD155 was localized in the cytoplasm and membrane of the tubular cells of PanIN2-3 lesions. **D.** The carcinoma cells showed intense staining for CD155 in the membrane. **E.**, **F.** The cancerous lesion ducts showed strong and diffuse staining for CD155 in the cytoplasm and membrane. **G.** CD155 expression in pancreatic cancer tissues was significantly higher than that in adjacent tissues.

Our results showed that the average percentage of CD155-positive cells in PC tissues and adjacent tissues was 92.64% (range, 40%-95%) and 39.52% (range, 0%-90%) respectively; the staining intensity score for CD155 was close to 3 in PC tissues and 1-3 in the adjacent tissues (Figure 2A-2F). As shown in Figure 2G, the IHC scores for CD155 in PC tissues were remarkably higher than those in adjacent tissues. Furthermore, the level of CD155 expression did not differ significantly according to patient clinicopathological features or tumor characteristics/tumor progression (Table 4), and CD155 was overexpressed in almost all the PC examples. These data indicate that CD226- and/or CD96-expressing NK cells have potential for the immune treatment of PC.

**Table 4:** CD155 expression according to patient characteristics and tumor progression indicators

Pancreatic cancer			
	NO. of patients	CD155	
		IHC scores	*P*
**Gender**			
Male	54	251.25±71.14	0.262
Female	34	248.82±52.43	
**Age**			
<65	50	248.15±67.21	0.651
≥65	38	253.16±60.89	
**Location**			
Head/Neck	62	243.30±71.33	0.104
Body/Tail	26	267.02±39.17	
**Tumor Size**			
≤2.5cm	15	248.94±63.04	0.527
>2.5cm	73	257.00±71.72	
**Histological grade**			
Well/Moderate	40	252.75±62.36	0.768
Poor	48	248.28±66.35	
**Nerve invasion**			
Absent	30	241.25±78.43	0.677
Present	58	255.00±55.71	
**Lymph node metastasis**			
Absent	49	257.45±47.04	0.647
Present	39	241.35±80.65	
**Blood vessel invasion**			
Absent	59	253.21±65.74	0.214
Present	29	243.79±61.69	

Data were expressed as means ± standard deviations (Mean ± SD).

## DISCUSSION

In our study, we evaluated the expression of two activating receptors, CD226 and CD96, and the inhibitory receptor TIGIT on NK cells from patients with PC by using FCM. CD226 and CD96 expression on NK cells was significantly decreased in PC, and their downregulation was correlated with the progression of PC. The findings therefore indicate that CD226 and CD96 could contribute to NK cell dysfunction and thereby induce PC progression and tumor immune escape.

CD226 is the most widely investigated activating receptor; it was initially described as an adhesion protein that promoted NK cell-induced killing of target cells. The binding of CD226 with CD112 or CD155 on the surface of transformed or infected cells promotes the synthesis of cytotoxic granules that induce the lysis of target cells [16, 17]. Furthermore, CD226 also enhances NK cell cytotoxicity by increasing the secretion of interferon-γ (IFN-γ) [18]. CD226-mediated NK cell function depends on the co-expression of LFA1 on NK cells and phosphorylation of Ser329 (a phosphorylation site on the intracellular domain of CD226) by protein kinase C [19, 20]. CD226-mediated NK cell dysfunction in malignancies, including PC, has been reported before (in a previous study on PC by the same authors) [21–23], but this study confirms the role of CD226 expression in PC in a bigger sample. The results were consistent with our previous results. Moreover, our results also demonstrated that lower CD226 expression could markedly promote the progression of PC.

CD96 is a rarely described surface receptor on NK cells, especially in cancers. Some studies suggest that CD96 not only enhances the adhesion between NK cells and target cells, but also increases the cytotoxicity of NK cells in humans; thus, its function seem to be similar to that of CD226 [13, 14]. However, in CD96^−/−^ mice, CD96 and CD226 were found to compete with each other for ligand binding, and CD96 was found to inhibit the *in vitro* and *in vivo* production of IFN-γ by NK cells [24]. This difference is probably attributable to inter-species differences [25]. Furthermore, the molecular structure of CD96 is different in mice and humans, which may also explain the difference in its function. An ITIM-like motif in the CD96 cytoplasmic domain could contribute to the generation of inhibitory signals in mice [26]. In our study, we found for the first time that CD96 expression was decreased in NK cells from PC patients and that it was closely correlated with PC progression. These findings indicate that CD96 could be considered as a potential activating receptor on NK cells from PC patients. To confirm the role of CD96, further studies on the function of CD96 in NK cells and the mechanism underlying CD96 downregulation in PC are required.

The inhibitory receptor TIGIT has been reported to inhibit the binding between CD226 and CD155 in a dose-dependent manner [27, 28]. TIGIT was reported to decrease NK cell-mediated killing of CD155-expressing cells in both humans and mice [14, 29, 30]. As reported in several articles, the expression of inhibitory receptors, such NKG2A [31] and KIR3DL1 [21], on NK cells usually increases in different cancers. However, in this study, we did not find differences between TIGIT expression in PC patients and healthy controls. Wang et al. reported that TIGIT is expressed preferentially on human NK cells but shows wide variation in its expression levels among healthy individuals [32], which could explain the findings of our study.

The use of immune therapy based on modified T or NK cells for malignancies has been gaining popularity. Enhancing CD226 and CD96 expression in NK cells would be an effective way of treating PC. However, this therapy relies on the higher expression of CD155 on PC cell surfaces. Overexpression of CD155 on the surface of PC cells was observed in our study, as well as another investigation by Satoshi et al. [33]. Therefore, NK cell therapy based on CD226 and CD96 expression should be explored in both *in vivo* and *in vitro* studies in the future.

## CONCLUSIONS

Significantly lower levels of CD226 and CD96 on NK cells from PC patients may indicate dysfunction of NK cells, and may therefore be indicators of the progression of PC. Therefore, CD226 and CD96 may participate in PC progression and immune escape induced by NK cell dysfunction. The findings of this study show that increasing CD226 and CD96 expression on NK cells may be a novel therapeutic strategy for the CD155-expressing PC.

## MATERIALS AND METHODS

### Patients and healthy controls

Blood samples used for flow cytometry (FCM) were obtained from patients (*n* = 90) with PC and healthy control individuals (*n* = 40). All peripheral blood samples from PC patients treated at the Pancreas Center of the First Affiliated Hospital of Nanjing Medical University were collected before surgery; none of the patients had undergone radiotherapy, chemotherapy or any other therapy before the surgery. Blood samples of the healthy controls were provided by the physical examination center of the First Affiliated Hospital of Nanjing Medical University. The main characteristics of the individuals enrolled are shown in Table 1. Tissue samples (88 pancreatic cancer tissues and 33 adjacent tissues) used for immunohistochemistry (IHC) were obtained from PC patients who had undergone surgical treatment at the Pancreas Center of the First Affiliated Hospital of Nanjing Medical University. Our study was approved by the ethics committee of the First Affiliated Hospital of Nanjing Medical University. Every patient and healthy control provided their informed consent for participation in this study.

### Antibodies and reagents

The anti-human CD3-PERCP, CD16-Brilliant Violet 510^TM^, CD56-Brilliant Violet 421^TM^, CD226-TITC, CD96-PE, TIGIT-APC, FITC mouse IgG1, PE mouse IgG1, and APC mouse IgG1 antibodies were all purchased from Biolegend (San Diego, CA, USA), as was the RBC lysis/fixation solution. The anti-human CD155 antibody was obtained from NOVUS (Littleton, CO, USA).

### Preparation of peripheral blood samples

Fresh blood sample (100 μl) was added into test tubes containing a specific marker. Following this, anti-human CD3-PERCP, CD16-Brilliant Violet 510^TM^, CD56-Brilliant Violet 421^TM^, CD226-TITC, CD96-PE, and TIGIT-APC antibodies were added into the tube. Anti-human CD3, CD16, and CD56 antibodies were used to identify NK cells (CD3-, CD16^+^ and/or CD56^+^). After incubation in the dark at room temperature for 15-20 min, 2 ml of 1× RBC lysis/fixation solution was added into the tube to remove the RBCs. The tube was further incubated in the dark at room temperature for 15 min, and the cells were then washed twice with PBS.

### FCM analysis

A cell subset located in the left lower quadrant (PBMCs) was selected based on the forward scatter (FSC) and side scatter (SSC) characteristics of the cells; this set was called “A” (Supplementary Figure 1). Then, the NK cell subset (CD3-, CD16^+^ and/or CD56^+^) was selected from set “A”. The cells in the NK subset were further analyzed for the presence of CD226, CD96, and TIGIT. Monoclonal antibodies with matched isotypes (FITC mouse IgG1, PE mouse IgG1, and APC mouse IgG1 antibodies) were used to exclude nonspecific fluorescence.

Multicolor FCM (Gallios; Beckman Coulter, Brea, CA, USA) and the Kaluza software (Beckman Coulter, Brea, CA, USA) were used to obtain and evaluate the data.

### IHC analysis

Two thick tissue sections (5 μm/section) were cut from the relevant tissue microarray with the assistance of pathologist Guo-Xin Song (Pathology Department, The First Affiliated Hospital of Nanjing Medical University) and labeled as “cancer tissues” or “adjacent tissues”. A total of 88 pancreatic cancer tissues and 33 adjacent tissues were enrolled in this study. All adjacent tissues enrolled in this study were obtained from part of patients which provided cancer tissues. After baking at 65°C for 90 min, the tissue sections were subject to immunohistochemical staining. Briefly, the tissue sections were consecutively deparaffinized in xylene and dehydrated in a graded alcohol series (ethyl alcohol, 95% alcohol, and 75% alcohol). In order to block endogenous peroxidase activity, the tissue sections were incubated in recently compounded 3% H_2_O_2_for 30 min. Then, the antigen was retrieved by preheating 10 mM sodium citrate buffer (pH 6.0) in a high-pressure steam boiler for 10 min. Non-specific binding was blocked by incubating the sections in phosphate-buffered saline supplemented with 10% normal goat serum at room temperature for 1 h. Next, the sections were incubated with anti-human CD155 antibody (NOVUS, Littleton, CO, USA) in PBS at 4°C overnight. After incubation with the primary antibody, the sections were treated with secondary antibodies at room temperature for 1 h, stained with diaminobenzidine for 5-10 min, and then counterstained with hematoxylin for 2 min.

The tissue sections were viewed separately by two experienced pathologists who were blinded to the clinicopathological data of the participants. A scoring method described previously was applied to evaluate the IHC staining. According to the percentage of CD155-positive cells, the following scores were assigned: 0 (0%), 1 (1%), 2 (2%) …… 99 (99%), 100 (100%). The staining intensity was scored as follows: 0 (negative staining), 1 (weak staining), 2 (moderate staining), and 3 (strong staining). Both scores were assigned independently by each pathologist, and they were also blinded to each other's assessment. The total score was obtained by multiplying the score for the percentage of positive staining with the staining intensity score.

### Statistical analysis

The Mann-Whitney *U*-test was used to analyze differences between groups. Pearson correlation analysis was used to assess the correlation between CD226 and CD96 expression. The association of protein expression with clinicopathologic features was analyzed by the Pearson χ2 test. All the statistical analyses were performed using SPSS 19.0 (SPSS Inc., Chicago, IL, USA). *P* < 0.05 was considered to indicate statistical significance.

## SUPPLEMENTARY MATERIAL FIGURE


