# Supplementary material for: Altered expression of CD226 and CD96 on natural killer cells in patients with pancreatic cancer
Source: Oncotarget. 2016 Sep 10;7(41):66586–94. doi: 10.18632/oncotarget.11953 (PMC5341822; doi:10.18632/oncotarget.11953)
Supplement: Supplementary file 1 [file oncotarget-07-66586-s001.pdf]

Altered expression of CD226 and CD96 on natural killer cells in patients with pancreatic cancer

Supplementary Material

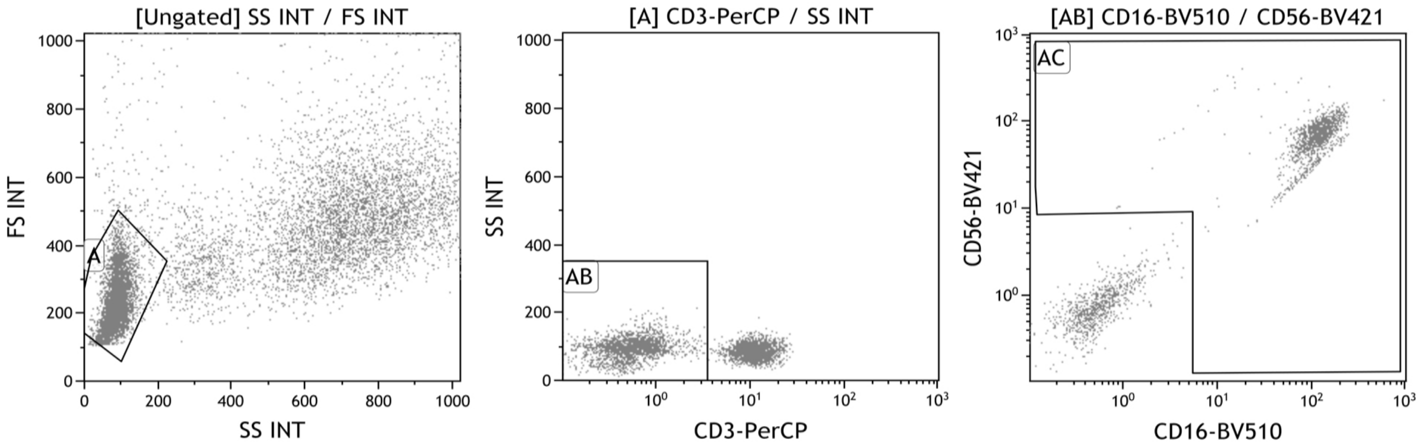

Supplementary Figure 1

According to forward scatter (FSC) and side scatter (SSC), a cell subset located in left lower quadrant (PBMCs) was selected from total cell subset and defined as gating “A”. And then, according to cells staining, another cell subset which detected as $CD3^-$, $CD16^+$ and/or $CD56^+$ (NK cells) was selected from gating “A” and defined as gating “AC”.
